# Comparison of Strategies for the Determination of Sterol Sulfates via GC-MS Leading to a Novel Deconjugation-Derivatization Protocol

**DOI:** 10.3390/molecules24132353

**Published:** 2019-06-26

**Authors:** Julia Junker, Isabelle Chong, Frits Kamp, Harald Steiner, Martin Giera, Christoph Müller, Franz Bracher

**Affiliations:** 1 Department of Pharmacy-Center for Drug Research, Ludwig-Maximilians University Munich, Butenandtstraße 5-13, 81377 Munich, Germany; 2 Biomedical Center (BMC), Metabolic Biochemistry, Ludwig-Maximilians University Munich, Feodor-Lynen-Strasse 17, 81377 Munich, Germany; 3 German Center for Neurodegenerative Diseases (DZNE), Feodor-Lynen-Strasse 17, 81377 Munich, Germany; 4 Leiden University Medical Center, Center for Proteomics and Metabolomics, Albinusdreef 2, 2300 RC Leiden, The Netherlands

**Keywords:** direct derivatization, solvolysis, sterol methoxime-trimethylsilyl ether

## Abstract

Sulfoconjugates of sterols play important roles as neurosteroids, neurotransmitters, and ion channel ligands in health and disease. In most cases, sterol conjugate analysis is performed with liquid chromatography-mass spectrometry. This is a valuable tool for routine analytics with the advantage of direct sterol sulfates analysis without previous cleavage and/or derivatization. The complementary technique gas chromatography-mass spectrometry (GC-MS) is a preeminent discovery tool in the field of sterolomics, but the analysis of sterol sulfates is hampered by mandatory deconjugation and derivatization. Despite the difficulties in sample workup, GC-MS is an indispensable tool for untargeted analysis and steroid profiling. There are no general sample preparation protocols for sterol sulfate analysis using GC-MS. In this study we present a reinvestigation and evaluation of different deconjugation and derivatization procedures with a set of representative sterol sulfates. The advantages and disadvantages of trimethylsilyl (TMS), methyloxime-trimethylsilyl (MO-TMS), and trifluoroacetyl (TFA) derivatives were examined. Different published procedures of sterol sulfate deconjugation, including enzymatic and chemical cleavage, were reinvestigated and examined for diverse sterol sulfates. Finally, we present a new protocol for the chemical cleavage of sterol sulfates, allowing for simultaneous deconjugation and derivatization, simplifying GC-MS based sterol sulfate analysis.

## 1. Introduction

The sulfoconjugates of sterols, also called sterol sulfates, are synthesized in vivo by conversion of the respective sterols by specific cytosolic sulfotransferase enzymes (SULT) [1,2]. These sterol sulfates are much more than just terminal stages of steroid metabolism and reservoir of their free analogues [2,3]. Several sterol sulfates are known to activate, modulate and inhibit specific enzymes and ion channels. For example, pregnenolone sulfate (**6**), dehydroepiandrosterone sulfate (**2**) and epipregnanolone sulfate are known to modulate neurotransmitter receptors like the γ-aminobutyric acid type A (GABA_A_) and the *N*-methyl-d-aspartate (NMDA) receptors [4,5]. Furthermore, epipregnanolone sulfate and pregnenolone sulfate (**6**) are activators of melastatin-like transient receptor potential (TRPM) ion channels [3,4]. Steroid sulfates can also bind to membrane-associated G-protein coupled receptors (GPCRs) and activate MAP kinase cascade or phospholipase C [6]. Amongst other functions, cholesteryl sulfate (**7**) interferes with blood coagulation by activating Factor XII and inhibiting the serine proteases thrombin and plasmin [7,8]. The balance between sulfatation and desulfatation is fundamental for the tissue distribution and function of sterols and its dysregulation is involved in many diseases [9]. For instance, pregnenolone sulfate (**6**) and dehydroepiandrosterone sulfate (**2**) have been reported to be decreased in the brains of Alzheimer’s disease (AD) patients [10]. Altered levels of pregnenolone sulfate (**6**) and several other sterol conjugates have also been found in the blood female AD patients [11], which might be related to an attenuated activity of SULT2A1 in the adrenal zona reticularis [12].

The analysis of sterol sulfates is hampered by the highly similar chemical structures of the sterol sulfates and their low abundance in biological samples. Several methodically different approaches are being applied in sterol sulfate analysis, the most common being radioimmunoassays (RIA), gas chromatography-mass spectrometry (GC-MS) and liquid chromatography-(tandem)mass spectrometry (LC-MS(/MS)). The major concerns about RIA are the need for using radioactive material as well as the low selectivity and possible cross-reactivity of similar analytes in addition to matrix effects [13]. In the last decade LC-MS(/MS) was the predominantly-used method for sterol conjugate analysis [14]. In contrast to GC-MS, as the gold standard of neutral cholesterol metabolites analysis [15], LC-MS(/MS) provides the possibility to analyze the non-volatile sterol conjugates without prior deconjugation [13,16,17]. Moreover, faster workup without deconjugation and/or derivatization and shorter run times of liquid chromatography makes it a high throughput method for targeted analysis ideally suited for clinical purposes [13,14]. Nevertheless, LC-MS(/MS) also has disadvantages such as limited chromatographic resolution and detection by electrospray ionization (ESI) mass spectra, that contain limited structural information due to low fragmentation rates [13,18]. In these aspects GC-MS cannot be replaced by LC-MS, since its high chromatographic resolution and the option for recording information-rich electron ionization (EI) mass spectra makes GC-MS a powerful tool for untargeted analyses and steroid profiling [13,16,17,18,19]. In particular, EI mass spectra of derivatives like sterol trimethylsilyl (TMS) ethers and methyloxime-trimethylsilyl (MO-TMS) ethers provide considerable structural information which can help to identify unknown steroidal analytes and can be used for suspected-target screening [18,20,21].

**Figure 1:**
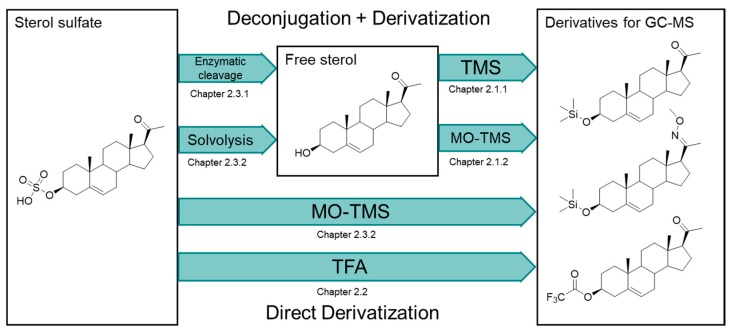
Strategies for sterol sulfate determination utilizing gas chromatography-mass spectrometry (GC-MS). “Two step” methods make use of a prior deconjugation step to form the free (unconjugated) sterol and a subsequent derivatization of the hydroxyl and, for methyloxime-trimethylsilyl (MO-TMS) derivatives, also the keto group. Direct derivatization refers to simultaneous cleavage of the sterol sulfate and derivatization. The deconjugation and derivatization strategies are shown with pregnenolone sulfate (**6**) here. TMS = derivatization to pregnenolone trimethylsilyl ether, MO-TMS = deconjugation/derivatization to pregnenolone methyloxime-trimethylsilyl ether, TFA = deconjugation/derivatization to pregnenolone trifluoroacetyl ester.

While these derivatization methods are well established for unconjugated steroids (free sterols including keto sterols, Figure 1) [20,22], there is no general procedure for the analysis of sterol sulfates using GC-MS. There are many different approaches published for deconjugation including enzymatic cleavage using sulfatases or chemical solvolysis, but an universally applicable method is lacking [23,24]. We discuss here in detail the most commonly used methods for deconjugation and derivatization for the GC-MS based analysis of sterol sulfates (Figure 1), and provide a significantly simplified procedure developed in the course of our investigations, allowing for simultaneous deconjugation and derivatization.

**Figure 2:**
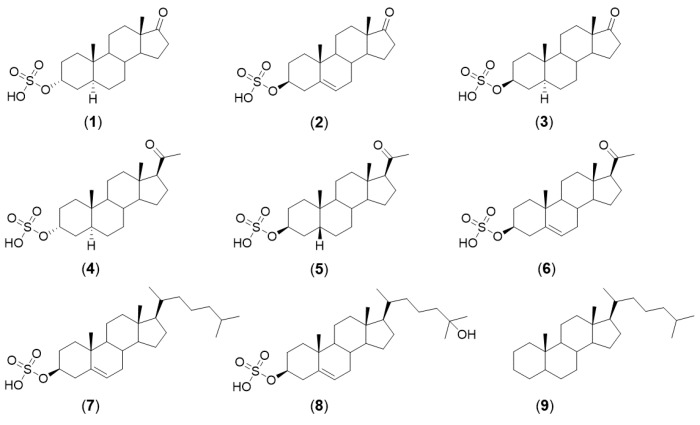
Overview of the model analytes: **1** androsterone sulfate, **2** dehydroepiandrosterone sulfate, **3** epiandrosterone sulfate, **4** allopregnanolone sulfate, **5** pregnanolone sulfate, **6** pregnenolone sulfate, **7** cholesterol sulfate, **8** 25-hydroxycholesterol sulfate, and **9** cholestane (internal standard, IS).

Furthermore, we present a comprehensive re-investigation of published methods demonstrating the scope and limitations of different derivatization procedures including direct acylation and formation of TMS and MO-TMS ethers. Additionally, we present a new protocol which allows the direct formation of MO-TMS derivatives from sterol sulfates, effectively combining sulfate ester cleavage and the formation of methyloximes (MO). The residual free hydroxyl groups can then be selectively silylated in a second step. The experiments were carried out with a representative collection of eight sterol sulfates with and without keto groups including 3α- and 3β-sterol sulfates and ∆^5^-unsaturated and saturated sterols. The structures of the model analytes are shown in Figure 2.

## 2. Results

### 2.1. Derivatization Strategies for Free Sterols (Deconjugated Sterol Sulfates) 

#### 2.1.1. Trimethylsilyl (TMS) Derivatives

The most popular derivatization method for sterols is the formation of sterol TMS ethers [22,25,26,27,28,29,30]. For this derivatization free hydroxyl groups are required, so in the case of sterol sulfates a prior deconjugation step is mandatory. Available deconjugation procedures are subject of Section 2.3. 

**Figure 3:**
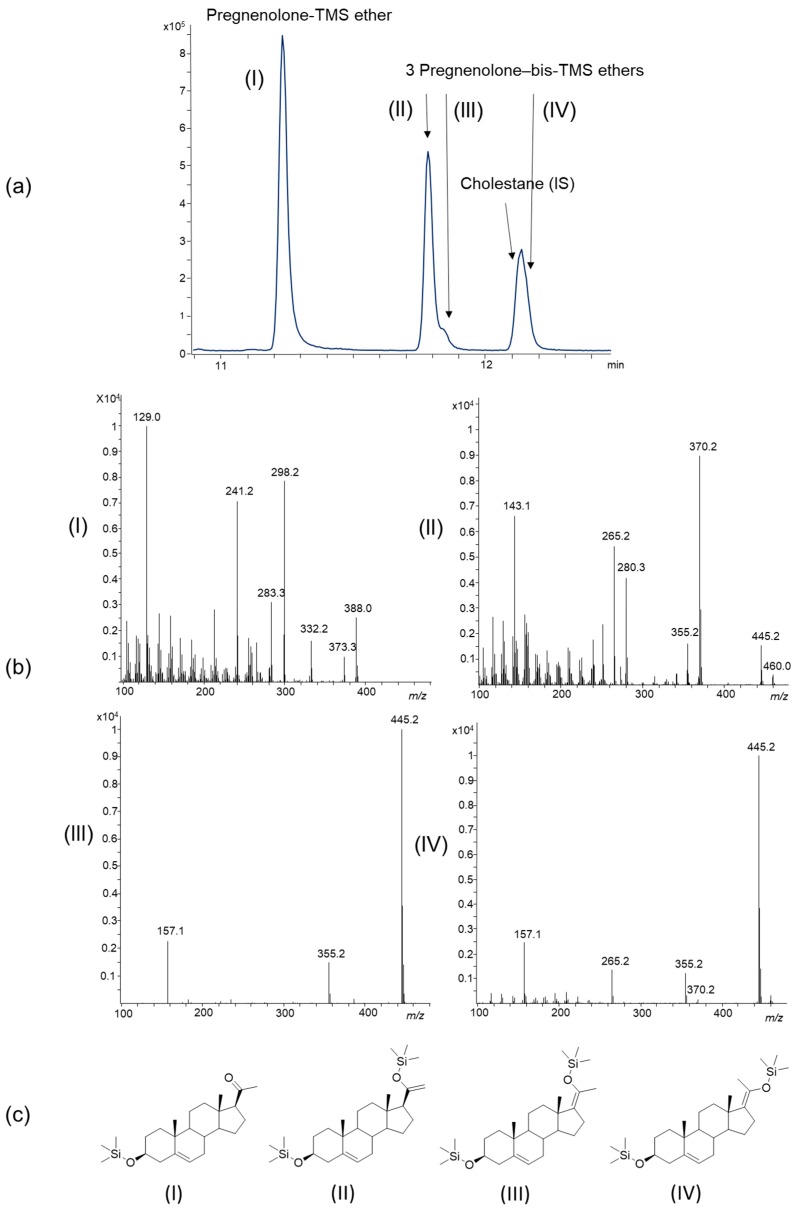
(**a**) Total ion chromatogram (TIC) of pregnenolone-TMS derivatives (containing cholestane (IS)). (**b**) Mass spectra of resulting pregenolone TMS ethers peaks (I)–(IV) after derivatization with *N*-methyl-*N*-trimethylsilyltrifluoroacetamide (MSTFA)/*N*-trimethylsilylimidazole (TSIM) (9:1). (**c**) Structures of pregnenolone-mono-TMS ether (I) and pregnenolone-bis-TMS ethers (II–IV) [33]; for chromatographic and mass spectral characteristics see Supplementary Table S1.

For silylation several reagents with different silyl donor abilities are available. To ensure a complete derivatization even of sterically hindered tertiary hydroxyl groups, the addition of a catalyst like *N*-trimethylsilylimidazole (TSIM) and trimethyliodosilane and/or an auxiliary base like pyridine is necessary [25]. An established silylation mixture for complete derivatization of secondary and tertiary hydroxyl groups even at room temperature is *N*-methyl-*N*-trimethylsilyltrifluoroacetamide (MSTFA) with 10% TSIM [20,31]. A known difficulty in TMS derivatization is the presence of keto groups, because the formation of artifacts (identified as enol TMS ethers) can be observed under these conditions [32]. We investigated the extent of the reported artifact formation for the exemplary keto sterol pregnenolone. The observed total ion chromatogram (TIC) in Figure 3 shows one peak (I) for pregnenolone with only one TMS ether (silylated 3-OH) and three (II–IV) artifacts corresponding to pregnenolone derivatives with an additional enol TMS ether. The plausible structures of these derivatives are shown in Figure 3c [33].

One attempt to avoid the formation of mixtures of mono- and bis-silylated products has been the application of a stronger silylating reagent which should enhance the enol TMS formation. For this purpose trimethyliodosilane can be used. This reactive reagent is generated in situ in a mixture of MSTFA and ammonium iodide. A reducing agent such as mercaptoethanol is further added in order to avoid undesired side reactions resulting from accidentally formed iodine [33,34,35,36,37,38]. This method requires much effort for optimization depending on the analytes of interest [36]. In addition also with this procedure artifacts can be observed, resulting from incorporation of mercaptoethanol [34,35]. In conclusion, silylation of keto sterols is cumbersome in most cases.

#### 2.1.2. Methyloxime-Trimethylsilyl (MO-TMS) Derivatives

**Scheme 1:**
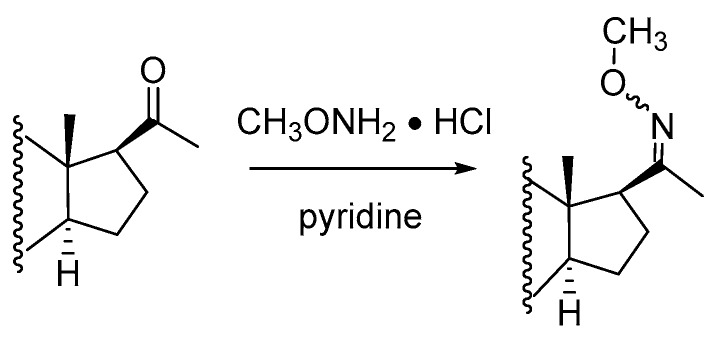
Example for derivatization of keto groups with *O*-methylhydroxylamine hydrochloride in pyridine [20].

The problematic (and frequently inevitable) enol TMS ether formation of keto sterols can be avoided with a two-step derivatization protocol. In this approach the keto groups are converted into methoxylamine (synonym: oxime methyl ether; MO) derivatives first, typically using 2% *O*-methylhydroxylamine hydrochloride (*m/v*) in pyridine (Scheme 1). In a second step the hydroxyl groups can be selectively transformed into TMS ethers using the methods described in Section 2.1.1 [20,32,34,39]. 

**Figure 4:**
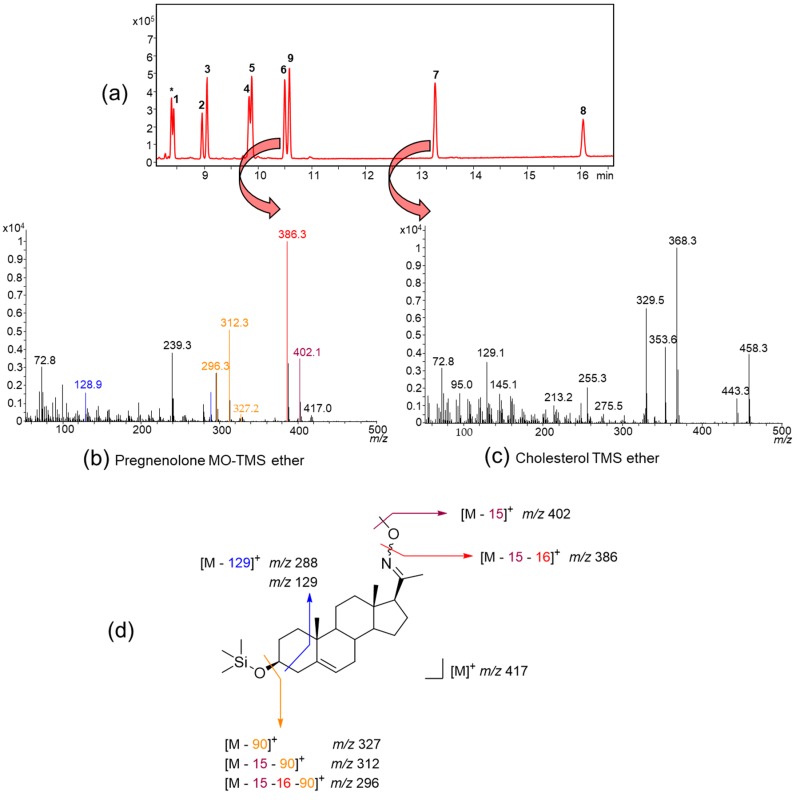
(**a**) Total ion chromatogram (TIC) of the eight sterol (MO-)TMS ethers and internal standard (IS). Analyzed sterols: **1** androsterone sulfate, **2** dehydroepiandrosterone sulfate, **3** epiandrosterone sulfate, **4** allopregnanolone sulfate, **5** pregnanolone sulfate, **6** pregnenolone sulfate, **7** cholesterol sulfate, **8** 25-hydroxycholesterol sulfate, and **9** cholestane (IS). * Impurity of silylating reagent; (**b**) mass spectrum of pregnenolone MO-TMS ether; (**c**) mass spectrum of cholesterol-TMS ether; (**d**) proposed fragmentations of pregnenolone MO-TMS ether according to literature [29]. For chromatographic and mass spectral characteristics see Supplementary Table S1.

However, with this method two isomeric MO derivatives (*syn*, *anti*) can be formed, which are partially or fully separated by GC giving two peaks with the same fragmentation patterns [32,40,41]. We were able to convert all exemplary keto sterol sulfates into their respective MO-TMS derivatives after solvolysis (see Section 2.3). The acquired chromatogram in Figure 4a shows only one peak for each sterol derivative and no additional peaks or peak shoulders due to *syn*-/*anti*-isomers of the MO residues were observed. With this procedure keto sterols (derived from sulfates **1**–**6**) and sterols without keto groups (derived from **7**, **8**) can be analyzed likewise.

The mass spectra of these derivatives provide much structural information. They show the molecular ion peaks and characteristic fragmentations, which can be seen in Figure 4b,c. As exemplarily shown for pregnenolone MO-TMS ether in Figure 4d the molecular ion [M]^+^ is observable, and the base peak *m/z* [M − 15 − 16]^+^ clearly indicates the fragmentation of the MO moiety. The ion *m/z* [M − 90]^+^ is typical for the loss of trimethylsilanol and the ions *m/z* [M − 129]^+^ as well as *m/z* 129 are characteristic for ∆^5^-sterol TMS ethers referring to the loss of trimethylsilanol from C-3 together with C-1, C-2 and C-3 [42]. 

### 2.2. Direct Deconjugation/Derivatization of Sterol Sulfates to Give Trifluoroacetyl (TFA) Derivatives

The problematic deconjugation step of sterol sulfates (for details see Section 2.3) can in certain cases be avoided if *O*-perfluoroacylation is chosen instead of TMS derivatization. The formation of perfluoroacyl derivatives is a fast and easy way to obtain volatile derivatives directly from sterol sulfates in one single operation. This method was first described by Touchstone and Dobbins [43] who used heptafluorobutyric anhydride (HFBA) in benzene to form the 3-*O*-acylated products directly from estriol sulfate and dehydroepiandrosterone sulfate (**2**). Also, Liere et al. [44] and Schumacher et al. [5] used successfully HFBA for the direct derivatization of **2** and **6** without prior sulfate deconjugation in one single step.

**Scheme 2:**
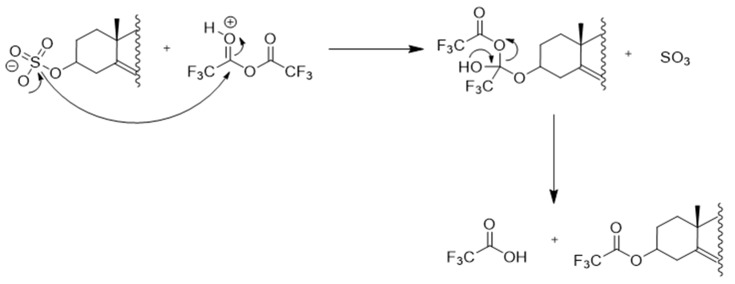
Mechanism for the acid-catalyzed reaction of ∆^5^-sterol sulfates with trifluoroacetic anhydride according to Murray and Baille [45].

Further investigations with different anhydrides, sterol sulfates and reaction conditions were performed by Murray and Baille [45], who observed that this direct derivatization protocol is limited to sulfates derived from ∆^5^-sterols and estrogens. They also showed that there is no need for using additional solvents like benzene, and demonstrated that the supplement of the auxiliary base pyridine, which is normally used to enhance the esterification of free sterols, even inhibits the reaction with sterol sulfates [45]. Complete derivatization of the ∆^5^-sterol sulfate dehydroepiandrosterone sulfate (**2**) was further obtained using trifluoroacetic anhydride (TFAA) without additional solvent reacted at 70 °C for 30 min. The authors [45] proposed an acid-catalyzed reaction which is shown in Scheme 2. 

**Figure 5:**
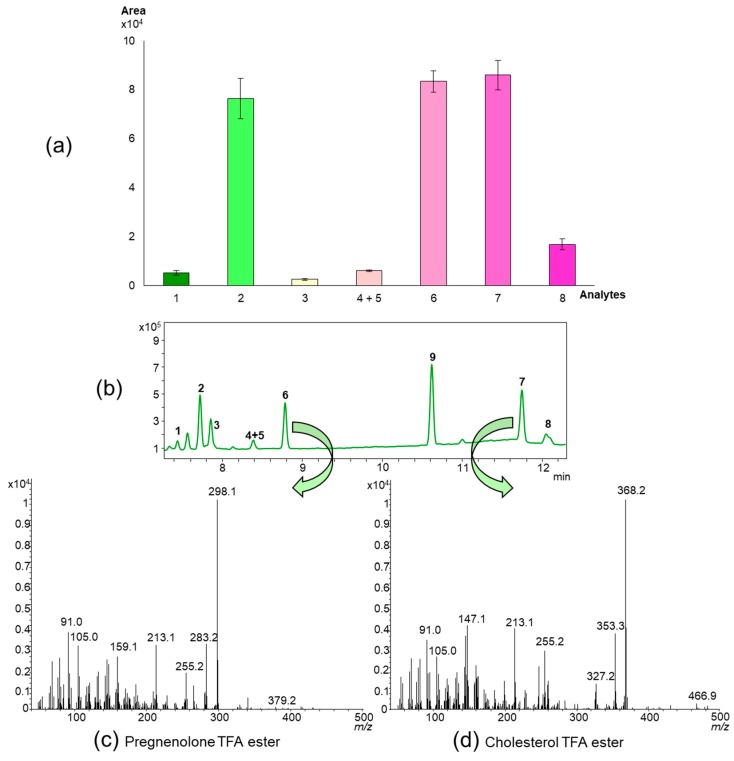
(**a**) Bar chart with mean base peak areas and standard deviations (n = 6) of the sterol TFA esters obtained by treatment of sterol sulfates **1**–**8** with trifluoroacetic anhydride. (**b**) Total ion chromatogram (TIC) of the eight sterol TFA esters and the internal standard, derived from: **1** androsterone sulfate, **2** dehydroepiandrosterone sulfate, **3** epiandrosterone sulfate, **4** allopregnanolone sulfate, **5** pregnanolone sulfate, **6** pregnenolone sulfate, **7** cholesterol sulfate, **8** 25-hydroxycholesterol sulfate, and **9** cholestane (IS). (**c**) Mass spectrum of pregnenolone TFA ester ([M]^+^ *m/z* 412). (**d**) Mass spectrum of cholesterol TFA ester ([M]^+^ *m/z* 482). For chromatographic and mass spectral characteristics see Supplementary Table S1.

We examined the scope of this direct derivatization protocol with the eight exemplary sterol sulfates shown in Figure 2. The results of this experiment are shown in Figure 5 and confirm the previously claimed limitation of this method to ∆^5^-sterol sulfates. The ∆^5^-unsaturated sterol sulfates **2**, **6**, and **7** showed good results while the saturated sterol sulfates **1**, **3**, **4**, and **5** did not undergo noteworthy conversion. An exception is the ∆^5^-unsaturated 25-hydroxycholesterol sulfate (**8**) whose TFA derivative was detected only in trace amounts (Figure 5a). The peak of analyte **8** in the chromatogram (Figure 5b) shows a peak shoulder and the corresponding mass spectra indicate an incomplete derivatization. The addition of pyridine is not useful in this case because it would inhibit the deconjugation of the sulfated hydroxyl group at C-3 at the same time [45].

Another weakness of this approach is the missing molecular ion of ∆^5^-sterol acyl derivatives [26,42,46,47] which is evident from the mass spectra shown in Figure 5c,d. This fact may lead to difficulties in identification of unknown compounds. Besides the missing molecular ion peak and the incomplete derivatization for some sterols, the residual TFA amounts in the samples lead to column bleeding and a shorter shelf life of the GC capillary column.

### 2.3. Strategies for Sterol Sulfate Deconjugation

#### 2.3.1. Enzymatic Cleavage of Sterol Sulfates

For the analysis of sterol sulfates as their corresponding TMS derivatives by GC-MS free hydroxyl groups of the unconjugated sterols are mandatory. Hence, an additional step for deconjugation is required. The enzymatic cleavage of sterol conjugates is a frequently used procedure especially in analysis of anabolic androgenic steroids in urine samples [23,24,48]. For glucuronides enzymatic cleavage utilizing the highly specific *E. coli* β-glucuronidase is the gold standard for steroid analysis in urine samples [23]. For the cleavage of sterol sulfates enzyme preparations from molluscs are commonly used, because these contain sulfatase activity beside β-glucuronidase activity. The most common preparations are from *Helix pomatia*, but also *Patella vulgata*, *Haliotis* spp. and *Ampullaria* are current sources [24]. These sulfatases are known to hydrolyze sulfates of 3β-hydroxy-∆^5^-sterols, 3β-hydroxy-5α-sterols, and 3α-hydroxy-5β-sterols, but fail to cleave 3α-hydroxy-5α-sterol sulfates [39,49]. Another known problem is the conversion and degradation of sterols especially by *Helix pomatia* preparations, which contain additional enzymes with various activities [24,25,28]. Due to these limitations there is no general procedure available for enzymatic cleavage of sterol sulfates. Gomes et al. [24] present several published procedures utilizing different enzymes, buffers and reaction conditions. We adopted the method described by Xu. et al. [50] with the difference that we used an aqueous solution of the sterol sulfates instead of a urinary sample. Under the described conditions (Section 5.3.4) we obtained only partial hydrolysis of dehydroepiandrosterone sulfate (**2**), a 3β-hydroxy-∆^5^-sterol sulfate, and epiandrosterone sulfate (**3**), a 3β-hydroxy-5α-sterol sulfate, with poor reproducibility. The other sterol sulfates in the experiment did not show any measurable hydrolysis. Variations of the buffer system (acetate buffer pH 7, phosphate buffer pH 5, 7, and 8) and reaction conditions (35 °C for 4 h and 20 h, 55 °C for 4 h and 20 h) did not improve our results. Hence, as optimization of the hydrolysis conditions is rather complex [48] and many sulfate conjugates (e.g., androsterone (**1**), a 3α-hydroxy-5α-sterol sulfate) are known to be resistant to enzymatic hydrolysis [39,49], this method seems not to be suitable for the untargeted analysis of sterol sulfates.

#### 2.3.2. Chemical Cleavage of Sterol Sulfates

**Scheme 3:**
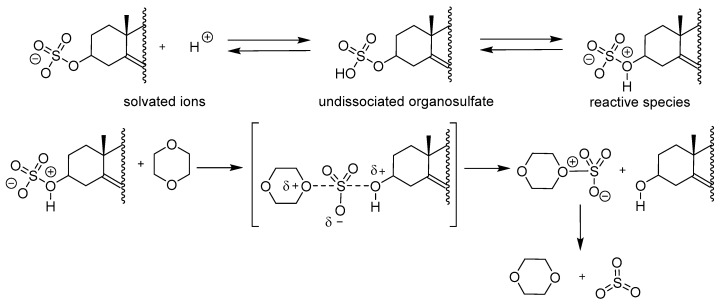
Mechanism of the acid-catalyzed solvolysis of sterol sulfates in 1,4-dioxane, as proposed by Burstein and Lieberman [55].

An alternative to the enzymatic hydrolysis is the chemical hydrolysis or solvolysis. Traditionally acidic hydrolysis at elevated temperatures was used for deconjugation of sterol sulfates. But the drastic conditions that are required for this hydrolysis including high amounts of mineral acid and refluxing, can lead to degradation or transformation of some sterols [51,52,53]. In turn, solvolysis under mild conditions is preferred and can be achieved by extracting the sterol sulfates from an acidified (with sulfuric acid) aqueous sample with ethyl acetate and storing this moist organic phase for 24 h at 39 °C [54] or with trimethylchlorosilane in methanol (methanolysis) [30]. The ability of oxygen-containing solvents, especially ethers, to cleave sterol sulfates in presence of minor amounts of water and acid was investigated in 1958 by Burstein and Lieberman [55]. They proposed an acid-catalyzed mechanism for the solvolysis (Scheme 3) in oxygen containing solvents, like 1,4-dioxane [55]. Having examined several published protocols, we found the solvolysis in 1,4-dioxane to be a particularly effective and mild method. It is applicable for both 3α- and 3β-sterol sulfates as well as for sulfates derived from saturated and unsaturated sterols [55,56].

**Figure 6:**
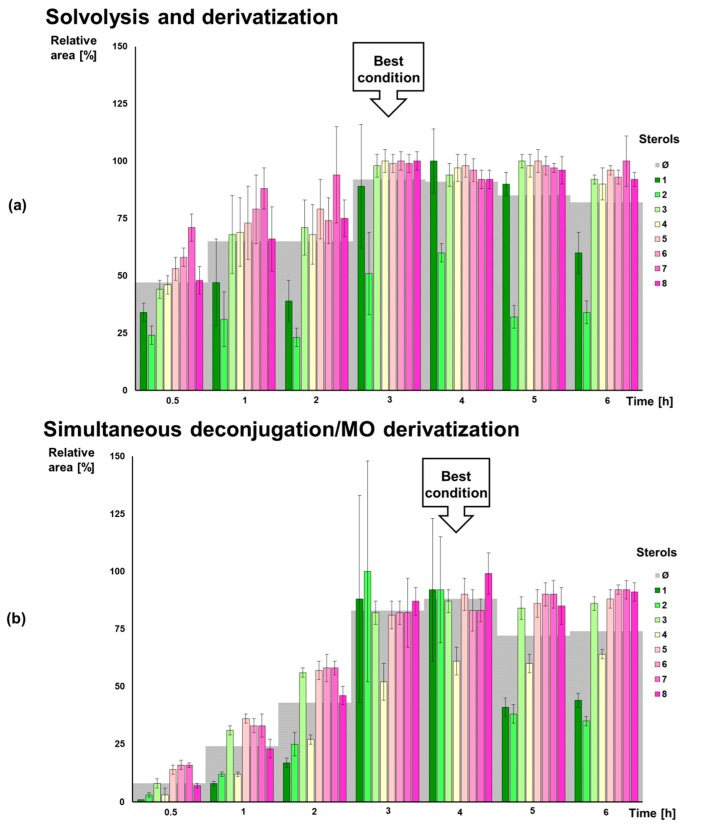
Determination of (keto-)sterol sulfates **1–8** as (MO-)TMS derivatives (**a**) with and (**b**) without previous sulfate solvolysis step (with 1% acetic acid in 1,4-dioxane). The indicated time refers to the duration of solvolysis prior to MO-TMS derivatization (for ”Solvolysis and derivatization”, (**a**)) or to the incubation with *O*-methylhydroxylamine solution (for ”Simultaneous deconjugation/MO derivatization”, (**b**)). The results obtained for each individual sterol sulfate under the different conditions are shown as relative peak areas [%] ± standard deviation (n = 6); the mean value of all steroids for every time point is shown in the background (grey), the best conditions for all tested sterol sulfates are marked as “Best condition”. The maximum recorded peak area for each sterol derivative within this experiment was set as 100%. Analyzed sterols: **1** androsterone sulfate, **2** dehydroepiandrosterone sulfate, **3** epiandrosterone sulfate, **4** allopregnanolone sulfate, **5** pregnanolone sulfate, **6** pregnenolone sulfate, **7** cholesterol sulfate, and **8** 25-hydroxycholesterol sulfate.

To examine the scope of solvolysis we modified a method published by Hutchins and Kaplanis [57] who applied 1% acetic acid in 1,4-dioxane under reflux overnight (here: ≤6 h, 100 °C; see Section 5.3.5.1). This solvolysis worked for every sterol sulfate in this experiment regardless of the configuration at C3 and presence of a ∆^5^-double bond. The experiments revealed the best reaction time for solvolysis was 3 h for the entire set of tested sterol sulfates. The optimum reaction times for solvolysis for individual sterol sulfates, shown in Figure 6a and Supplementary Table S2, vary between 3 h and 4 h. The solvolyzed sterol sulfates were measured as their MO-TMS derivatives (two-step derivatization as described in Section 2.1.2). 

Further experiments surprisingly revealed a possibly new form of chemical cleavage. Sterol sulfate deconjugation was found to be a side effect of the first derivatization step, the methyloxime (MO) formation of the keto groups. We examined scope and efficiency of this new method for simultaneous cleavage and MO derivatization of sterol sulfates in additional experiments. To this end, eight sterol sulfates **1**–**8** (Figure 2) were incubated with *O*-methylhydroxylamine solution for different times (0.5 h–6 h; see Section 5.3.5.2) without previous solvolysis, then silylated, and the results were compared with the results of the solvolysis approach. This comparison is shown in Figure 6b, and Supplementary Table S2. In conclusion, we found that the acidic solvolysis step is dispensable for all investigated sterol sulfates. Optimal results for all analytes under investigation, using our new simultaneous deconjugation/MO derivatization protocol, were obtained after 4 h incubation with *O*-methylhydroxylamine solution. The optimum conditions of this simultaneous deconjugation/MO derivatization method for each individual sterol sulfate, shown in Figure 6b and Supplementary Table S2, vary between 3 h and 6 h. Two criteria were employed for evaluation of optimal conditions, on the one hand the relative peak area was taken as indicator for the degree of deconjugation, on the other hand the standard deviation (SD) should be as small as possible. 

Figure 6a shows that solvolysis is a reliable method which achieves the best results for most of the tested sterol sulfates (100% is the best result achieved for individual sterols, not the recovery). The disadvantage of solvolysis is the additional workup step, because derivatization including methyloxime formation for 0.5 h, if keto sterols are analyzed, and silylation has to be performed in addition to the solvolysis step. This extra deconjugation procedure can be avoided in the approach with simultaneous deconjugation/MO derivatization. In this case, incubation for 4 h achieves the best results for most of the tested sterol sulfates. The peak areas achieved under these conditions are similar to those obtained with solvolysis with the advantage of less workup efforts. 

Which method should be preferred is dependent on the target analytes. If sterols without keto groups are analyzed solely, a simplified approach with solvolysis and subsequent silylation is advisable. If keto sterols are determined it depends on the particular sterols, for example for dehydroepiandrosterone sulfate (**2**) better results can be achieved with the simultaneous deconjugation/MO derivatization protocol, whereas allopregnanolone sulfate (**4**) can be cleaved with solvolysis more effectively. 

## 3. Discussion

We investigated the scope and limitations of most of the commonly used procedures including direct acylation and formation of TMS and MO-TMS ethers. The advantages and disadvantages of these methods are summarized in Table 1.

Surprisingly, we found that in the course of the methoximation of keto sterol sulfates, originally intended only to protect their keto groups as MO derivatives for avoiding undesired enol silylation in the subsequent silylation of the 3-hydroxy groups (see Section 2.1.2), that sterol sulfates were as well cleaved upon treatment with the *O*-methylhydroxylamine reagent. To the best of our knowledge, this reaction has not yet been utilized in the analysis of sterol sulfates before. Only scarce evidence on this type of organosulfate cleavage has been published before, and previous investigations were performed only with aryl [58] and methyl sulfates [59,60]. Most likely, this exceptional reactivity of methoxylamine is due to the so-called α-effect [59,61], leading to strongly enhanced nucleophilicity of the NH_2_ group, even enabling this reagent to cleave organosulfates under uncommon nucleophilic attack at the S-atom. This novel sample pretreatment allows for an unprecedented, short and easy-to-perform derivatization of keto sterol sulfates involving both organosulfate deconjugation and ketone methoximation under relatively mild reaction conditions. Subsequent silylation of liberated hydroxyl groups provides suitable derivatives for GC-MS analysis. Hence, this new deconjugation/derivatization protocol represents a considerable progress in the analysis of keto sterol sulfates. Our present investigations on the chemical behavior of sterol sulfates provided further useful evidence for the analysis of sterol sulfates. 

## 4. Conclusions

The aim of the present work was to find the best deconjugation/derivatization strategy for the analysis of sterol sulfates by GC-MS. As expected, there is no single best method for deconjunction and derivatization of sterol sulfates. Depending on the nature of the analyte of interest, the methods have individual strengths and weaknesses (Section 2.3.2, Table 1). For the targeted determination of known (∆^5^-)sterol sulfates an especially fast workup employing direct perfluoroacylation can be the method of choice. But one of the biggest advantages of GC-MS is its strength as discovery tool for unexpected sterols. For this untargeted approach workup procedures are necessary, that are not limited to a subset of sterol sulfates. In addition, these workup procedures should form derivatives with characteristic mass spectra. Both our new protocol for simultaneous deconjugation/MO-derivatization followed by TMS derivatization and the protocol for acidic solvolysis followed by MO-TMS derivatization meet these requirements.

## 5. Materials and Methods

### 5.1. Materials and Reagents

All consumables were from VWR (Ismaning, Germany). Derivatization reagents trifluoroacetic anhydride (TFAA), 1-(trimethylsilyl)imidazole (TSIM), and *N*-methyl-*N*-trimethylsilyltrifluoroacetamide (MSTFA) were from Macherey-Nagel (Düren, Germany). Deionized water was prepared with an in-house ion-exchanger. 1,4-Dioxane and methyl *tert*-butyl ether (M*t*BE) were distilled before use. β-Glucuronidase/sulfatase from *Helix pomatia* type HP-2, 5α-cholestane (≥97%), pregnenolone (>98%), pregnenolone sulfate sodium salt (>98%), and cholesteryl sulfate sodium salt (>99%) were purchased from Sigma-Aldrich (Schnelldorf, Germany). Dehydroepiandrosterone sulfate sodium salt (>99%) and 25-hydroxycholesteryl sulfate sodium salt (>99%) were from Avanti Polar Lipids (Alabaster, AL, USA). All other sterol sulfate sodium salts were from Steraloids (Newport, RI, USA). All other reagents and solvents were purchased in HPLC grade or in pro analysis quality from Sigma-Aldrich (Schnelldorf, Germany).

### 5.2. Instruments and Equipment

Gas chromatography (GC) was performed on a Varian 3800 gas chromatograph coupled to a Saturn 2200 ion trap from Varian (Darmstadt, Germany). The autosampler was from CTC Analytics (Zwingen, Switzerland) and the split/splitless injector was a Varian 1177 (Darmstadt, Germany). Instrument control and data analysis were carried out with Varian Workstation 6.9 SP1 software (Darmstadt, Germany) and Agilent MassHunter Workstation Software package B.08.00 (Santa Clara, CA, USA). An Agilent HP-5-ms capillary column (Santa Clara, CA, USA) of 30 m length, 0.25 mm i.d., and 0.25 µm film thickness was used at a constant flow rate of 1.4 mL/min. Carrier gas was helium 99.999% from Air Liquide (Düsseldorf, Germany). The inlet temperature was kept at 300 °C and injection volume was 1 µL with splitless time 1.0 min. The initial column temperature was 50 °C and was held for 1.0 min. Then temperature was ramped up to 250 °C with 50 °C/min. Then the sterols were eluted at a rate of 5 °C/min until 310 °C (hold time 3 min). Total run time was 20 min. Transfer line temperature was 300 °C and the ion trap temperature was 150 °C. The ion trap was operated with electron ionization (EI) at 70 eV in scan mode (*m*/*z* 50–650) with a solvent delay of 6.3 min.

### 5.3. Methods

A stock solution containing androsterone sulfate (**1**), dehydroepiandrosterone sulfate (**2**), epiandrosterone sulfate (**3**), allopregnanolone sulfate (**4**), pregnanolone sulfate (**5**), pregnenolone sulfate (**6**), cholesterol sulfate (**7**), 25-hydroxycholesterol sulfate (**8**), and cholestane (**9**) as internal standard (IS) with a concentration of 10 µM of each analyte in ethyl acetate was prepared. Substance structures are shown in Figure 1.

#### 5.3.1. TMS Derivatives by Direct Silylation

Pregnenolone (2 µg) and cholestane (1 µg, IS) was silylated with 50 µL of a mixture of MSTFA and TSIM (9:1) at room temperature for 30 min. After the addition of 950 µL methyl *tert*-butyl ether (M*t*BE) the sample was analyzed as described above by GC-MS.

#### 5.3.2. Acidic Deconjugation and Formation of MO-TMS Derivatives 

An aliquot of the stock solution containing 10 nmol of each sterol sulfate and IS was transferred into an autosampler vial and the solvent (ethyl acetate) was evaporated under a stream of nitrogen. Deconjugation was performed in 1,4-dioxane with 1% acetic acid (*v*/*v*) for 3 h (see Section 5.3.5.1). Subsequently, the sample was evaporated to dryness under a stream of nitrogen. The dry residue was derivatized with 100 µL 2% *O*-methylhydroxylamine hydrochloride in pyridine (*m*/*v*) at 80 °C for 30 min. This reaction time is sufficient for a complete derivatization of the keto groups. Then the sample was diluted with 400 µL water and the sterols were extracted with 2 × 1000 µL M*t*BE. The combined organic phases were transferred into a new autosampler vial and evaporated to dryness under a stream of nitrogen. Then the residue was silylated with 50 µL of a mixture of MSTFA and TSIM (9:1) at room temperature for 30 min. After addition of 950 µL M*t*BE the sample was analyzed by GC-MS.

#### 5.3.3. TFA Derivatives by Direct Deconjugation/Derivatization

An aliquot of the stock solution containing 10 nmol of each sterol sulfate and IS was transferred into an autosampler vial (*n* = 6) and evaporated to dryness under a stream of nitrogen. Fifty microliters of trifluoroacetic anhydride (TFAA) was added to the residue. The vial was closed and stored at 70 °C for 30 min, then the volatiles were evaporated under a stream of nitrogen. The residue was dissolved in 1000 µL M*t*BE and analyzed by GC-MS.

#### 5.3.4. Enzymatic Cleavage of Sulfates and Derivatization

An aliquot of the stock solution containing 10 nmol of each sterol sulfate and IS was transferred into an autosampler vial (*n* = 6) and was evaporated to dryness under a stream of nitrogen. The residue was diluted in 0.5 mL water and 0.5 mL buffer containing β-glucuronidase/sulfatase from *Helix pomatia* type HP-2 was added [50]. The closed vial was stored at 37 °C for 20 h. Then the sample was extracted with 2 × 1000 µL M*t*BE. The combined organic phases were transferred into a new autosampler vial and evaporated to dryness under a stream of nitrogen. The residue was silylated with 50 µL of a mixture of MSTFA and TSIM (9:1) at room temperature for 30 min. After addition of 950 µL M*t*BE the sample was analyzed by GC-MS. The acquired peak area for each sterol was compared to the area obtained by solvolysis (see Section 5.3.5.1) followed by TMS derivatization. The obtained data are listed in Table 1.

#### 5.3.5. Chemical Cleavage of Sulfates and Derivatization

##### 5.3.5.1. With Acidic Deconjugation (Solvolysis)

An aliquot of the stock solution containing 10 nmol of each sterol sulfate and IS was transferred into an autosampler vial (*n* = 6) and the solvent (ethyl acetate) was evaporated under a stream of nitrogen. For solvolysis 500 µL of 1,4-dioxane with 1% acetic acid (*v/v*) was added and the vial was closed tightly. The mixture was stored at 100 °C for different periods of time (0.5–6 h, Figure 6 and Supplementary Table S2). Then the sample was evaporated to dryness under a stream of nitrogen. The residue was derivatized with 100 µL 2% *O*-methylhydroxylamine hydrochloride in pyridine (*m/v*) at 80 °C for 30 min. Then the sample was diluted with 400 µL water and the sterols were extracted with 2 × 1000 µL M*t*BE. The combined organic phases were transferred into a new autosampler vial and evaporated to dryness under a stream of nitrogen. Then the residue was silylated with 50 µL of a mixture of MSTFA and TSIM (9:1) at room temperature for 30 min. After addition of 950 µL M*t*BE the sample was analyzed by GC-MS.

##### 5.3.5.2. With Deconjugation/Methoximation with *O*-Methylhydroxylamine

An aliquot of the stock solution containing 10 nmol of each sterol sulfate and IS was transferred into an autosampler vial (*n* = 6) and the solvent (ethyl acetate) was evaporated under a stream of nitrogen. Simultaneous deconjugation/MO derivatization was achieved by addition of 100 µL 2% *O*-methylhydroxylamine hydrochloride in pyridine (*m*/*v*) directly to the neat sterol sulfates (*n* = 6). The vial was stored at 80 °C for different periods of time (0.5–6 h, Figure 6 and Supplementary Table S2). Then the sample was diluted with 400 µL water and the sterols were extracted with 2 × 1000 µL M*t*BE. The combined organic phases were transferred into a new autosampler vial and evaporated to dryness under a stream of nitrogen. Then the residue was silylated with 50 µL of a mixture of MSTFA and TSIM (9:1) at room temperature for 30 min. After addition of 950 µL M*t*BE the sample was analyzed by GC-MS.

## Figures and Tables

**Table 1:** Overview of derivatization methods for analysis of sterol sulfates.

Analyzed by GC-MS as	Advantages	Disadvantages
**TMS ether** **(Section 2.1.1 and Section 5.3.1)**	Fast and easy workup Mass spectra with molecular ion and characteristic fragmentation pattern	Prior deconjugation step afforded (e.g., solvolysis) Artifact formation with keto groups possible
**MO-TMS ether** **(Section 2.1.2, Section 2.3.2, Section 5.3.2 and Section 5.3.5.2)**	Mass spectra with molecular ion and characteristic fragmentation pattern Simultaneous deconjugation/MO-derivatization of sterol sulfates possible No artifacts (apart from possible *syn*- and *anti*-isomers of the MO group) in presence of keto groups	Time consuming workup with two step derivatization and additional clean up step
**TFA ester** **(Section 2.2 and Section 5.3.3)**	Fast and easy workup Direct derivatization of sterol sulfates possibleShort GC run times	Direct derivatization is limited to estrogens and ∆^5^-sterol-3-sulfates Derivatization of additional free hydroxyl groups could be problematic (e.g. 25-hydroxycholesterol sulfate) Residual TFA leads to column bleeding Mass spectra do not show a molecular ion

## Supplementary Materials

Table S1: Gas chromatography-mass spectrometry data for the eight model sterol sulfates. Table S2: Determination of sterol sulfates as MO-TMS derivatives with and without solvolysis.
