# Supplementary material for: Comparison of Strategies for the Determination of Sterol Sulfates via GC-MS Leading to a Novel Deconjugation-Derivatization Protocol
Source: Molecules. 2019 Jun 26;24(13):2353. doi: 10.3390/molecules24132353 (PMC6651411; doi:10.3390/molecules24132353)
Supplement: Supplementary file 1 [file molecules-24-02353-s001.pdf]

Table S1. Gas chromatography-mass spectrometry (GC-MS) data for the derivatives obtained from the eight model sterol sulfates **1** – **8** using different deconjugation/derivatization protocols; chemical formulae of the sterol sulfates; TFA: trifluoroacetyl ester; TMS: trimethylsilyl ether; MO-TMS: methyloxime-trimethylsilyl ether; relative retention times (RRT) related to internal standard cholestane (**9**); bold *m*/*z* value: base peak; * predominantly (2 × TFA); [a] analyzed as TMS ether (since no keto group present for MO formation); [b] no derivatization possible.

No.	Compound	Chemical formula	Relative Retention Time (RRT)			Characteristic ions [*m*/*z*]		
			TFA	TMS	MO-TMS	TFA	TMS	MO-TMS
**1**	Androsterone sulfate	$C_{19}H_{30}O_5S$	0.701	0.759	0.796	386<br>368<br>**342**	347<br>**272**<br>215	391<br>360<br>**270**
**2**	Dehydroepiandrosterone sulfate	$C_{19}H_{28}O_5S$	0.728	0.804	0.846	**270**<br>255<br>121	360<br>270<br>**129**	389<br>358<br>**268**
**3**	Epiandrosterone sulfate	$C_{19}H_{30}O_5S$	0.732	0.813	0.855	386<br>368<br>**342**	**347**<br>272<br>215	391<br>**360**<br>270
**4**	Allopregnanolone sulfate	$C_{21}H_{34}O_5S$	0.790	0.873	0.929	396*<br>267<br>**215**	**300**<br>285<br>215	404<br>**388**<br>100
**5**	Pregnanolone sulfate	$C_{21}H_{34}O_5S$	0.790	0.882	0.934	**396***<br>267<br>215	375<br>**300**<br>285	404<br>**388**<br>298
**6**	Pregnenolone sulfate	$C_{21}H_{32}O_5S$	0.827	0.926	0.992	**298**<br>283<br>213	388<br>298<br>**129**	402<br>**386**<br>312
**7**	Cholesterol sulfate	$C_{27}H_{46}O_4S$	1.106	1.257	1.257	**368**<br>353<br>255	458<br>**368**<br>329	458[a]<br>**368**<br>329
**8**	25-Hydroxycholesterol sulfate	$C_{27}H_{46}O_5S$	1.134	1.516	1.516	**366**<br>351<br>245	456<br>271<br>**131**	456[a]<br>271<br>**131**
**9**	Cholestane (IS)	$C_{27}H_{48}$	1.000	1.000	1.000	357[b]<br>262<br>**217**	357[b]<br>262<br>**217**	357[b]<br>262<br>**217**

Table S2. Determination of (keto-)sterol sulfates **1** - **8** as (MO-)TMS derivatives with and without previous sulfate solvolysis step (with 1% acetic acid in 1,4-dioxane). The indicated time refers to the duration of solvolysis prior to derivatization (MO-TMS) (upper row "Solv. + Deriv.") or to the duration of the simultaneous deconjugation/MO derivatization (lower row "Deconjug./MO"). The results obtained for each individual sterol sulfate under the different conditions are shown as relative peak areas [%] ± standard deviation (n = 6); optimum conditions for all tested sterol sulfates are shown in the last two rows **Ø 1 – 8**; in bold: the best conditions for each sterol sulfate; in red: optimum method for all tested sterol sulfates. The maximum recorded peak area for each sterol derivative within this experiment was set as 100% (Note: The values presented here are *not* recoveries). Analyzed sterols: **1** androsterone sulfate, **2** dehydroepiandrosterone sulfate, **3** epiandrosterone sulfate, **4** allopregnanolone sulfate, **5** pregnanolone sulfate, **6** pregnenolone sulfate, **7** cholesterol sulfate, **8** 25-hydroxycholesterol sulfate.

**Sterol sulfate**	**Method**	**Incubation time**						
		**0.5 h**	**1 h**	**2 h**	**3 h**	**4 h**	**5 h**	**6 h**
**1**	Solv. + Deriv.	34 ± 4	47 ± 19	39 ± 9	89 ± 27	**100 ± 14**	60 ± 5	60 ± 9
	Deconjug./MO	0 ± 0	8 ± 1	17 ± 2	88 ± 45	**92 ± 31**	41 ± 4	44 ± 3
**2**	Solv. + Deriv.	24 ± 4	31 ± 12	23 ± 4	51 ± 18	**60 ± 4**	32 ± 5	34 ± 5
	Deconjug./MO.	3 ± 1	12 ± 1	24 ± 5	100 ± 48	**92 ± 23**	38 ± 4	35 ± 2
**3**	Solv. + Deriv.	44 ± 4	68 ± 17	71 ± 12	**98 ± 5**	94 ± 5	100 ± 3	92 ± 2
	Deconjug./MO.	8 ± 2	31 ± 2	56 ± 2	82 ± 5	**87 ± 5**	84 ± 5	86 ± 3
**4**	Solv. + Deriv.	46 ± 4	69 ± 15	68 ± 13	**100 ± 5**	97 ± 6	98 ± 5	90 ±7
	Deconjug./MO	3 ± 1	12 ± 1	27 ± 2	52 ± 8	61 ± 6	**60 ± 4**	64 ± 2
**5**	Solv. + Deriv.	53 ± 5	73 ± 16	79 ± 13	**99 ± 4**	98 ± 5	100 ± 5	96 ± 2
	Deconjug./MO	14 ± 2	36 ± 2	57 ± 4	81 ± 6	**90 ± 7**	86 ± 6	88 ± 4
**6**	Solv. + Deriv.	58 ± 4	79 ± 15	74 ± 10	**100 ± 4**	96 ± 5	98 ± 4	93 ± 3
	Deconjug./MO	13 ± 2	38 ± 3	62 ± 6	92 ± 5	**100 ± 9**	93 ± 5	95 ± 2
**7**	Solv. + Deriv.	71 ± 6	88 ± 9	94 ± 21	**99 ± 4**	92 ± 4	97 ± 2	100 ± 11
	Deconjug./MO	16 ± 1	33 ± 5	58 ± 3	82 ± 15	83 ± 5	90 ± 6	**92 ± 4**
**8**	Solv. + Deriv.	48 ± 6	66 ± 14	75 ± 8	**100 ± 4**	92 ± 4	96 ± 6	92 ± 3
	Deconjug./MO	7 ± 1	23 ± 4	46 ± 4	87 ± 6	**99 ± 9**	85 ± 8	91 ± 4
**Ø 1 - 8**	Solv. + Deriv.	47 ± 4	65 ± 15	65 ± 11	**92 ± 9**	91 ± 6	85 ± 4	82 ± 5
	Deconjug./MO	8 ± 1	24 ± 2	43 ± 3	83 ± 17	**88 ± 12**	72 ± 5	74 ± 3
